# Knowledge, awareness and attitudes about cervical cancer among women attending or not an HIV treatment center in Lao PDR

**DOI:** 10.1186/1471-2407-14-161

**Published:** 2014-03-06

**Authors:** Chanvilay SICHANH, Fabrice QUET, Phetsavanh CHANTHAVILAY, Joeffroy DIENDERE, Vatthanaphone LATTHAPHASAVANG, Christophe LONGUET, Yves BUISSON

**Affiliations:** 1 Institut de la Francophonie pour la Médecine Tropicale (IFMT), Vientiane, Lao PDR; 2 UMR 1094 (Université de Limoges/Inserm/CHU de Limoges) Neuroépidémiologie Tropicale (NET), Limoges, France; 3 University of Health Sciences, Vientiane, Lao PDR; 4 Department of Infectious Diseases, Mahosot hospital, Vientiane, Lao PDR; 5 Fondation Mérieux, Lyon, France

**Keywords:** Cervical cancer screening, HIV/AIDS, Knowledge, Awareness, Attitude, Lao PDR

## Abstract

**Background:**

Cervical cancer is the first female cancer in Lao PDR, a low-income country with no national screening and prevention programs for this human papillomavirus (HPV) associated pathology. HIV-infected women have a higher risk of persistent oncogenic HPV infection.

The purpose of this study was to determine the knowledge, awareness and attitudes about cervical cancer among Lao women attending or not an HIV treatment center, in order to understand if this attendance had offered an opportunity for information and prevention.

**Methods:**

A cross-sectional case–control survey was conducted in three provinces of Lao PDR, Vientiane, Luang Prabang and Savannakhet. Cases were 320 women aged 25 to 65, living with HIV and followed in an HIV treatment center. Controls were 320 women matched for age and place of residence, not attending an HIV treatment center.

**Results:**

Cases had a greater number of sexual partners and used condoms more often than controls. Only 36.6% of women had consulted a gynecologist (47.5% among cases and 25.6% among controls, p < 0.001) and 3.9% had benefited from at least one Pap smear screening (5.6% cases and 2.2% controls, p = 0.02). The average knowledge score was 3.5 on a 0 to 13 scale, significantly higher in cases than in controls (p < 0.0001). Despite having a lower education level and economic status, the women living with HIV had a better knowledge about cervical cancer and were more aware than the controls of the risk of developing such a cancer (35.9% vs. 8.4%, p = 0.0001). The main source of information was healthcare professionals. The main reasons for not undergoing Pap smear were the absence of symptoms and the default of medical injunction for cases, the lack of information and ignorance of screening usefulness for controls.

**Conclusion:**

In Lao PDR, routine consultation in HIV treatment centers is not enough harnessed to inform women of their high risk of developing cervical cancer, and to perform screening testing and treatment of precancerous lesions. Implementing this cost-effective strategy could be the first step toward a national prevention program for cervical cancer.

## Background

Cervical cancer is the second most common cancer in women worldwide, with over 90% of cases occurring in developing countries [1]. A persistent infection with a high risk oncogenic Human papillomavirus (HR-HPV) is involved in almost all cases [2]. HPV infection is very common in young women with early sexual activity, with a peak before 25 years, usually without clinical consequence [3]. In nearly 10% of cases, this infection persists and is associated after 5 to 10 years with lesions that may regress, remain stable or progress to a higher grade and invasive cancer. Evolution of cervical intraepithelial neoplasia (CIN) to invasive cancer is slow, about 10 to 20 years for an immunocompetent woman [4]. This slow progression allows an effective secondary prevention based on screening and treatment of precancerous lesions, using cervical cytological testing according Papanicolaou (Pap smear or Pap test), visual inspection of the cervix with 3–5% acetic acid (VIA), or more recently HPV DNA testing.

Immunosuppression, especially due to human immunodeficiency virus (HIV) infection, is a predisposing factor for persistent infection with HR-HPV [5] and the development of squamous intraepithelial lesions (SIL) [6]. High HIV viral loads and low CD4 counts are associated with a higher risk of HR-HPV infection and cervical abnormalities [7]. The risk of recurrence or progression of cervical lesions is 4–5 times higher in women living with HIV [8]. Infection with one of the 15 HR-HPV genotypes is significantly more common in HIV-infected women [9], while the distribution of low-risk oncogenic HPV is not affected by HIV status [10]. This reflects a higher propensity of HR-HPV in determining persistent infections [11].

Before the era of antiretroviral therapy, the life expectancy of HIV-infected women was too short for a systematic cervical cancer screening program to demonstrate effectiveness [12]. Today, despite highly active antiretroviral therapy (HAART) guided by CD4 count, the probability of developing invasive cervical cancer remains high and stable in this population at risk [13,14]. Thus, the follow-up of women on antiretroviral therapy offers an opportunity for cervical cancer screening in resource-poor countries [15,16]. A prospective study with a follow-up over 10 years on a cohort of 1760 HIV-infected and 472 non HIV-infected women showed that HIV-positive women who benefitted from regular screening had no greater risk of developing invasive cancer than HIV-negative women [17].

Laos has a female population of 1.79 million aged 15 and older. Cervical cancer is the most common cancer in women with a 15.8 per 100,000 estimated crude incidence. It is also the second leading cause of cancer death in women, but the primary cause between 15 and 44 years [18]. As in most low-income countries, there are no national screening and prevention programs for cervical cancer in Lao PDR and awareness of women is still very low.

The prevalence of HIV infection in Lao PDR is relatively low compared to neighboring countries, around 0.2% among adults aged 15–49 in the general population. In 2011, the number of women living with HIV was estimated at 5,263 and 50.9% of eligible women were receiving HAART under the National HIV/AIDS Program supported by the Global Fund [19]. Patients are followed in seven treatment centers, two in the capital of Vientiane, the other five in the provinces of Luang Prabang, Savannakhet, Luang Namtha, Bokeo and Pakse. There is currently no routine screening program for cervical cancer in women living with HIV in Laos. As the primary prevention of cervical cancer by vaccination is not effective in women already infected by HPV, the only recourse is the secondary prevention through early detection and treatment of SIL [13]. Attending HIV treatment centers is an opportunity to educate women about the risks and the value of cytological screening. The aim of this study was to determine the knowledge and awareness of Lao women about cervical cancer and to evaluate the impact of medical care for HIV-infected women on their risk awareness and prevention behaviors.

## Methods

### Study type

A cross-sectional case–control survey was conducted by interviews in three provinces selected for the presence of HIV treatment centers: Vientiane capital (1,320 patients followed, including 581 women), Luang Prabang in the north (138 patients including 62 women) and Savannakhet in the south (1171 patients, including 844 women).

### Study population

The sample size was calculated to be 640 (320 cases and 320 controls) on the assumption that in the group followed in the HIV treatment centers (cases), the relative percentage of women aware of cervical cancer was 10% higher than in the control group, with a precision of 10%, an alpha risk of 5%, and a 90% power.

The cases were HIV-positive women, aged 25 to 65, living in the province and regularly monitored in the HIV treatment centers. Controls were women matched on age and dwelling place, not attending HIV treatment centers. Women unable to respond to questions or not willing to participate in the study were excluded.

From the 560 HIV-infected women who met the inclusion criteria in the three provinces (30 in Luang Prabang, 190 in Vientiane and 340 in Savannakhet), 320 were randomly selected. For each case, a control with the same age (± 1 year), living in the same district was randomly drawn from the census of residents.

### Data collection

A standardized questionnaire was used to collect information upon knowledge, awareness and attitudes of women about the risk of cervical cancer and its prevention. The questionnaire consisted of four sections: (i) socio-demographic data, (ii) risk behaviors for sexually transmitted infections (STIs) and immune status for HIV-infected women, (iii) knowledge on cervical cancer and its prevention, (iv) attitudes towards prevention and screening (see Additional file 1). All the questions were tested before the survey. The level of knowledge was assessed on 13 questions, possible answers being “yes/no/do not know.” Ambiguous responses were clarified using complementary open questions. For each respondent, the overall knowledge scores were calculated as the sum of correct answers, values ranging from 0 up to 13 [20]. Open-ended questions were also asked in the fourth section to specify the reasons for the non-use of neither gynecological consultation nor screening.

The cases were recruited during their visit to the HIV treatment center. Seropositivity and immune status were checked on their medical records. The questionnaire was administered anonymously and confidentially by a female physician (CS), student of IFMT especially trained in investigation. The interviews took place in a private room for an average time of 15 to 20 minutes. The cases not regularly monitored by the center were visited at home. Controls were recruited in the same district of residence than matched cases and were interviewed at home using the same standardized questionnaire. After the interview, the women were given counselling on cervical cancer screening.

### Data analysis

Data entry was performed using SPSS Version 19 software, and analyses of variables performed with STATA version 11. We used the Chi 2 test or Fisher exact to compare qualitative variables, Student’s t test and ANOVA for quantitative variables with normal distribution, Wilcoxon and Kruskal-Wallis tests for quantitative variables with non-normal distribution. Factors associated to a score of knowledge about cervical cancer better than 3 (dependant variable) were assessed by logistic regression. All independent variables with a p-value under 0.25% in univariate analyses were considered and included in the initial model in a unconditional way. The final model was obtained using backward elimination i.e. the progressive elimination of non-significant factors by decreasing order of significance. Results of univariate and multivariate analyses (final model) were merged and presented in the same table. For all analyses, a p-value under 0.05 was considered as significant.

### Ethical clearance

Women enrolled in this study received a clear description of the study objectives and the working methods. They were informed that the investigation concerned sexuality and addressed some information upon their privacy, but that all data would remain anonymous and confidential. They were free to accept or decline the interview and signed an informed consent form in case of acceptance. The study received approval from the National Ethics Committee of the Ministry of Health of Lao PDR and was carried out with the authorization of the HIV treatment centers in the three provinces.

## Results

The theoretical sample size of 640 women was achieved in the three sites: 434 in Savannakhet (67.8%), 146 in Vientiane (22.8%) and 60 in Luang Prabang (9.4%). Only three women refused the interview. At each site, 50% of recruited women were cases and 50% were matched controls.

### Socio-demographics

The mean age of these 640 women was 36.2 ± 8 years (range 25–63), 80% were married and almost all were Buddhist (96%). Occupations were mainly farmer (32.7%) and shopkeeper (27.2%). The significant differences between cases and controls were the number of children, the level of education and the average monthly income, all higher in the control group (Table 1).

**Table 1:** Demographic characteristics, risk factors and behaviours of women followed in the HIV treatment centres (cases) and matched controls

***Demographic characteristics***	**Total**	**Cases**	**Controls**	**p**
	**N = 640 (%)**	**N = 320 (%)**	**N = 320 (%)**	
**Mean age**	36.2 ± 8	36.3 ± 8.1	36.1 ± 7.9	0.70
**Marital status**				0.10
Single	30 (4.7)	10 (3.1)	20 (6.3)	
Bride/concubine	513 (80.2)	256 (80)	257 (80.3)	
Divorced/widow	97 (15.2)	54 (16.9)	43 (13.4)	
**Average number of children**	2.3 ± 1.5	2.0 ± 1.5	2.6 ± 1.4	<0.0001
**Religion**				0.40
Buddhist	614 (95.9)	305 (95.3)	309 (96.5)	
Animistic	26 (4.1)	15 (4.6)	11 (3.4)	
**Education level**				0.002
Illiterate	48 (7.5)	26 (8.1)	22 (6.9)	
Primary	337 (52.7)	188 (58.8)	149 (46.6)	
Secondary	255 (39.8)	106 (33.1)	149 (46.6)	
**Occupation**				0.20
Housewife	110 (11.2)	53 (16.6)	57 (17.8)	
Civil servant	50 (7.8)	20 (6.3)	30 (9.4)	
Shopkeeper	174 (27.2)	82 (25.6)	92 (28.8)	
Farmer	209 (32.7)	113 (35.3)	96 (30)	
Factory woman	88 (13.8)	45 (14.1)	43 (13.4)	
Hairdresser	5 (0.8)	3 (0.9)	2 (0.6)	
Sex worker	4 (0.6)	4 (1.3)	0	
**Average income/month (US dollars)**	85.3 ± 69.2	79.2 ± 46.1	91.4 ± 85.8	0.01
**Province**				1.00
Luang Prabang	60 (9.4)	30 (9.4)	30 (9.4)	
Savannakhet	434 (67.8)	217 (67.8)	217 (67.8)	
Vientiane	146 (22.8)	73 (22.8)	73 (22.8)	
***Risk factors and behaviours***				
**Average age at first intercourse**	18.6 ± 2.7	18.6 ± 2.9	18.7 ± 2.4	0.80
**Average number of sexual partners**	2.5 ± 6.3	3.6 ± 8.7	1.5 ± 1.3	<0.0001
**Current use of condom**				<0.001
- Never	338 (52.8)	81 (25.3)	257 (80.3)	
- Sometimes	61 (20.2)	32 (13.4)	29 (46.0)	
- Often	48 (15.9)	33 (13.8)	15 (23.8)	
- Always	193 (63.9)	174 (72.8)	19 (30.2)	
Current method of contraception^*^				
- Oral or injectable contraception	104 (16.3)	25 (7.8)	79 (24.7)	<0.001
- *Coitus interruptus*	28 (4.4)	5 (1.6)	23 (7.2)	0.001
- Intra-uterine device	11 (2.5)	3 (1.1)	8 (4.6)	0.10
- Tubal ligation	4 (0.9)	1 (0.3)	3 (1.7)	0.30
**Smoking**	82 (12.8)	57 (17.8)	25 (7.8)	<0.001
**Have consulted a gynaecologist**	234 (36.6)	152 (47.5)	82 (25.6)	<0.001
**Have had ≥ 1 Pap smear screening**	25 (3.9)	18 (5.6)	7 (2.2)	0.02

*including condom use.

### Risk factors

The average age at first intercourse was 18.6 ± 2.7 years, almost identical in both groups. Women living with HIV had more sexual partners, used condoms more often and consumed more tobacco than controls. However, the use of contraceptive methods other than condoms, including the combined pill, was more common among controls. Overall 234 women (36.6%) had consulted a gynecologist and 25 (3.9%) had had one Pap smear test. These two practices were significantly more frequent among women living with HIV (Table 2). The number of gynecological consultations was related to education level (p = 0.01) and amount of income (p = 0.001), but these two factors had no significant influence on the use of the Pap smear.

**Table 2:** Knowledge and attitudes of cases (women followed in the HIV treatment centres) and matched controls

***Knowledge about sexually transmitted infections (STIs) and cervical cancer (CC)***	**Total**	**Cases**	**Controls**	**p**
	**N = 640**	**N = 320**	**N = 320**	
	**(%)**	**(%)**	**(%)**	
**Able to name:** at least 1 STI	417 (65.2)	290 (90.6)	127 (39.7)	0.0001
1 STI	207 (32.3)	151 (47.1)	58 (18.1)	0.0001
2 STI	122 (19.1)	79 (24.7)	43 (13.4)	0.0001
3 STI	68 (10.6)	47 (14.7)	21 (6.6)	0.001
4 STI	20 (3.1)	13 (4.1)	5 (1.6)	0.05
**Have heard about:**				
- CC	344 (53.8)	177 (55.3)	167 (52.2)	0.40
- Human papillomavirus (HPV)	37 (5.8)	14 (4.4)	23 (7.2)	0.10
- HPV vaccination	65 (10.2)	41 (12.8)	24 (7.5)	0.02
**Knowledge scores** (0 to 13) ≤ 3	351 (54.8)	146 (45.6)	205 (64.1)	0.0001
> 3	289 (45.2)	174 (54.4)	115 (35.9)	
**Knowing someone with CC**	118 (18.4)	41 (12.8)	77 (24.1)	0.0002
**Knowing that:**				
- CC is a serious condition	318 (49.7)	186 (58.1)	132 (41.3)	0.0001
- CC is caused by HPV infection	20 (3.1)	10 (3.1)	10 (3.1)	1.00
- Multiple sexual partners are a risk factor of CC	224 (35)	179 (55.9)	45 (14.1)	0.01
**Able to name at least:**				
- One risk factor for CC	49 (7.7)	29 (9.1)	20 (6.3)	0.10
- One screening method of CC	17 (2.7)	11 (3.4)	6 (1.9)	0.20
- One prevention method of CC	105 (16.4)	51 (15.9)	54 (16.9)	0.70
**Mentioned prevention methods of CC:**				
- Drugs	41 (6.4)	14 (4.4)	27 (8.4)	0.05
- Vaccination	156 (24.4)	46 (14.4)	110 (34.4)	0.0001
- Annual gynaecological exam	294 (45.9)	184 (57.5)	110 (34.4)	0.0001
- Vaccination and annual gynaecological exam	149 (23.1)	83 (25.9)	66 (20.3)	0.08
**Sources of information on CC:**				
- Health professionals	197 (30.8)	127 (39.7)	70 (21.9)	0.0001
- Medias	82 (12.8)	28 (8.8)	54 (16.9)	0.002
- Family and relatives	65 (10.6)	22 (6.9)	43 (13.4)	0.006
**Sources of information on HPV vaccination**				
- Health professionals	45 (7.0)	29 (9.1)	16 (5.0)	0.04
- Medias	16 (2.5)	9 (2.8)	7 (2.2)	0.60
- Family and relatives	4 (0.6)	3 (0.9)	1 (0.3)	0.60
**Attitudes with respect to the risk of CC**				
**Believing:**				
- That CC is a common disease in Laos	323 (50.5)	183 (57.2)	140 (43.8)	0.0007
- To be herself at risk of developing CC	142 (22.2)	115 (35.9)	27 (8.4)	0.0001
**Regarding as risk factors for CC:**				
- HIV infection	413 (64.5)	236 (73.8)	177 (55.3)	0.0001
- Unprotected intercourse	560 (87.5)	293 (91.6)	267 (83.4)	0.001
- Multiple sexual partners	574 (89.7)	297 (92.8)	277 (86.6)	0.01
- Smoking	265 (41.5)	125 (39.1)	140 (43.9)	0.20
**Wishing:**				
- National recommendations for HPV vaccination	582 (90.9)	288 (90.0)	294 (91.9)	0.40
- To be vaccinated if an affordable HPV vaccine is offered	568 (88.8)	288 (90.0)	280 (87.5)	0.30

### Clinical and immunological status of women living with HIV

Among the 320 HIV infected women, 181 (56.6%) had a history of one or more opportunistic infections and 289 (90.3%) were on antiretroviral therapy, regularly followed in the majority of cases. The follow-up in HIV treatment centers was planned once a month in Vientiane and Luang Prabang and once every two months in Savannakhet. The CD4 cell count was less than 200/mm^3^ in 162 (50.6%) patients.

### Knowledge about cervical cancer

For all the women surveyed, the average knowledge score noted from 0 up to 13 was 3.5 ± 2.1 (range: 0–11). Unrelated to age, the highest scores were recorded among single women, of secondary education level, civil servant, with a monthly income greater than 85 USD and living in Vientiane province. Scores were significantly higher among women living with HIV than in controls (p < 0.0001), except among unmarried and women living in the province of Luang Prabang.

In multivariate analysis after logistical regression, five variables were independently found associated with a score of knowledge upon cervical cancer higher than 3: occupation, province, education level, matrimonial status and HIV status (Table 3). Civil servants were more than 9 times more aware about cervical cancer than housewives (reference value). Women attending care in Vientiane were almost 4 times more aware than those in Luang Prabang. Those who had reached at least the secondary school education level were 3.2 times more aware than illiterates ones. Women divorced or widowed were 3.1 times more aware than single ones. Finally, people living with HIV were found 2.8 times more aware than controls.

**Table 3:** Logistical regression: factors associated with a score of knowledge upon cervical cancer higher than 3

**Variables**	**Univariate analysis**			**Multivariate analysis**		
	**cOR**	**CI**_**95%**_	**p**	**aOR**	**CI**_**95%**_	**p**
**Occupation**						
Civil servant (ref: housewife)	8.18	[3.50; 19.09]	<0.001	9.23	[3.52; 24.0168]	<0.001
Shopkeeper (ref: housewife)	1.36	[0.84; 2.20]	0.2			
Farmer (ref: housewife)	0.99	[0.61; 1.58]	0.9			
Factory woman (ref: housewife)	0.93	[0.53; 1.66]	0.8			
**Province**						
Vientiane (ref: Savannakhet)	1.82	[1.25; 2.66]	0.002			
Vientiane (ref: Luang Prabang)	1.98	[1.07; 3.64]	0.03	3.77	[1.78; 7.99]	0.001
**Education level**						
Primary (ref: illiterate)	1.43	[0.74; 2.77]	0.3			
At least secondary (ref: illiterate)	3.47	[1.77; 6.78]	<0.001	3.20	[1.53; 6.70]	0.002
**Marital status**						
Bride/concubine (ref: single)	3.06	[1.37; 6.81]	0.006			
Divorced/widow (ref: single)	2.59	[1.08; 6.22]	0.03	3.09	[1.53; 6.70]	0.002
**HIV status**						
Case (ref: control)	2.12	[1.53; 2.94]	<0.001	2.78	[1.95; 3.96]	<0.001
**Monthly income (US $)**						
At least 85$ (ref: lower than 85$)	1.96	[1.42; 2.70]	<0.001			

*Ref*, Reference; *cOR*, Crude Odd Ratio; *CI*_*95%*_, 95% Confidence Interval; *aOR*, Adjusted Odd Ratio.

A slight majority of respondents (53.8%) had heard of cervical cancer and 18.4% knew someone with the disease. Most felt that it is a serious condition but only 40 (6.3%) were aware of the role of HPV. Women living with HIV had a significantly higher level of knowledge about STIs in general, the severity of cervical cancer, the risk related to the number of sexual partners and prevention methods (Table 2).

Health professionals were the main source of information, especially for women living with HIV (p = 0.0001), whereas controls rather knew the disease by the media (p = 0.002) or their family (p = 0.006).

### Attitudes towards the risk of cervical cancer

Half of the women interviewed believed that cervical cancer is a common disease in Laos, but only 22% felt themselves at risk, this proportion being higher among women living with HIV (p = 0.0001). Similarly, the risk factors associated with HIV infection, unprotected sex and multiple partners were better perceived by cases than by controls. In both groups, a large majority of women wished that a preventive vaccine for cervical cancer be recommended by the government and planned to benefit themselves from it if its cost was moderate (Table 2).

Among women living with HIV, the main reason for non-completion of Pap smear screening was the absence of symptoms (p < 0.0001) and the lack of injunction from the doctor (p = 0.04). Other reasons were the lack of information on screening and its usefulness, more often alleged by the controls (Table 4).

**Table 4:** Reasons for not undergoing a Pap smear screening among cases (women followed in the HIV treatment centres) and matched controls

**Reasons**	**Total**	**Cases**	**Controls**	**p**
	**N = 615**	**N = 302**	**N = 313**	
	**(%)**	**(%)**	**(%)**	
No symptoms	203 (33.0)	139 (46.0)	64 (20.4)	0.0001
Never heard of screening	160 (26.0)	64 (21.2)	96 (30.7)	0.003
Believing it is not necessary	92 (14.9)	33 (10.9)	59 (18.8)	0.003
Not enough available	51 (8.3)	17 (5.6)	34 (10.9)	0.01
Not prescribed by the doctor	33 (5.4)	22 (7.3)	11 (3.5)	0.04
Fear to be too expensive	22 (3.6)	13 (4.3)	9 (2.9)	0.30
No risk factor	19 (3.1)	9 (1.5)	10 (3.2)	0.80
Fear of painful sampling	17 (2.8)	0 (0)	17 (5.4)	0.0001
Screening centre too far	7 (1.1)	4 (1.3)	3 (0.9)	1.00
Fear of an abnormal result	5 (0.8)	0 (0)	5 (1.6)	0.06
Shame or embarrassment	4 (0.7)	1 (0.3)	3 (0.9)	0.60
Absence of sexual activity	2 (0.3)	0 (0)	2 (0.6)	0.50

## Discussion

Early detection of pre-cancerous lesions is universally recognized as the most effective method of preventing cervical cancer. However low-income countries, where this cancer has the highest incidence, still face many challenges to establish national prevention programs [21]. Thus in Laos the screening coverage for women aged 18 to 69 years was estimated at 2.2%, 5.2% in urban areas and 1.4% in rural areas [18]. The level of women’s knowledge about risk factors and prevention of cervical cancer is a major determinant to undergo screening tests [22]. This survey is the first one conducted among Lao women aged 25 to 65 on knowledge, awareness and attitude about cervical cancer. It has been conducted in three different regions of the country and involved a representative sample of the female population living with HIV and a matched control group.

The first result of this study is that the general level of knowledge of the women interviewed is very poor, most being unable to mention any STI, never having heard of HPV and knowing no way to prevent cervical cancer. Knowledge mean scores are even lower than those found in Chiang Mai, Thailand, in a survey upon 402 sex workers. This difference could be explained by the fact that Thai STI services also offer a Pap screening for exposed women [20]. The best knowledge scores were observed in women with a secondary education level and earning more than 85 US dollars per month. Similar results are found in different studies conducted in low-income countries such as Cameroon [22], Nigeria [23], Tanzania [21], and Ethiopia [24]. In addition, the civil servant status which gives access to social insurance in Laos and the residence in Vientiane capital, where access to care is easier than in the provinces, are factors independently associated with a higher level of knowledge.

Almost half of women have heard of cervical cancer and consider it as a serious and common disease in Laos. But being aware does not necessarily correspond to a correct understanding of the disease [25]. Although a large majority cites unprotected sex and multiple sex partners as main risk factors, only 6% know the causal link between HR-HPV infection and cervical cancer. This rate was 14% among sex workers in Chiang Mai [20] and reached 64% in a multicenter survey in the United States [26], but in both cases, the questionnaire offered a multiple-choice question for this item.

The other interesting aspect highlighted by the survey is that HIV infected women have a significantly higher level of knowledge than their controls, although their average level of education is lower. The same observation was made in a study conducted in the United States on 1588 women, including 71% HIV positive, showing that women living with HIV had better understanding of the prevention of cervical cancer than HIV-negative women [26]. In our survey, this difference is observed mainly on issues related to sexually transmitted nature of the infection, HIV and HPV sharing common routes of transmission. There is also evidence that women living with HIV are more likely to use condoms and to consult a gynecologist than controls.

However, knowledge about risk factors for cervical cancer, screening methods and means of prevention are similarly poor in both groups. Few women consider themselves at risk of developing cervical cancer. Although this awareness is four times higher among HIV-infected women (35.9%) than among controls (8.4%), it does not lead to a greater practice of smear screening. Indeed, only 5.6% of HIV infected women had undergone a Pap test. A similar proportion (5%) was found in a study conducted among 300 women attending an HIV treatment center in Lagos, Nigeria, in the absence of national program for cervical cancer screening [27]. In addition, half of HIV-infected women in our study had a CD4 count below 200/mm^3^. This immune deficiency is associated with an increased risk of cervical dysplasia, but has also been identified as a risk factor for non-adherence to Pap smear program in a US survey [28].

It is noteworthy that the absence of symptoms was the reason given by one third of women and 46% of women infected with HIV for not performing testing. The same reason, advanced by 67% of sex workers in a study in Thailand, reflects a total ignorance of the natural history of cervical cancer and the principle of Pap screening, hence the need to strengthen the education of these highly exposed women [20].

The finding that nearly 90% of women in both groups hope national recommendations for vaccination against HPV and would agree to receive it should be interpreted with caution. This wish ignores that vaccination is only for girls who have not started their sexual life, while commercial vaccines do not protect against all types of HPV circulating in Southeast Asia [29].

Health personnel represent the main source of information for women living with HIV, before media and family or friends entourage. Regular attendance of HIV treatment centers has an obvious impact on the awareness of the risk of cervical cancer, but it still has no effect on screening behavior. Several reasons could be advanced. First, physicians responsible for monitoring and treatment of HIV patients do not have sufficient training to counsel their patients on the prevention of cervical cancer. A survey conducted in Karachi, Pakistan, amongst interns and nursing staff in tertiary care hospitals showed that only 40% knew the Pap smear as a screening tool for cervical cancer [30]. Second, the HIV post-test counseling and the therapeutic monitoring are not used to discuss the screening of cervical cancer. As in many low-income countries, this screening is not part of the routine management of HIV-infected women and health practitioners in HIV treatment centers do not receive proper training [27]. Third, the screening capacities in the Lao health system do not yet allow responding to a systematic demand for all women at risk. In addition, the cost of Pap smear, around 10 U.S. dollars, is a deterrent for a majority of women who do not have social insurance.

Our study has some limitations like all surveys by questionnaire covering information about private life. Response biases through default of sincerity are hard to avoid completely. Regarding the cases, it was not possible to determine the anteriority and regularity of consultations in HIV treatment centers. In addition, the HIV-negative status of controls could not be confirmed, this position being assumed on the non-attendance of HIV treatment centers.

The main result of this survey is the highlight of a wide ignorance on cervical cancer and its prevention amongst Lao women. It urges to implement information campaigns on a national scale specifically targeting women aged 25 to 65. This health education must take into account the ability to understand and the cultural characteristics of women. It is necessary to improve the communication skills to effectively convey prevention messages to women less educated [31]. This is the case of women belonging to various ethnic minorities in Southeast Asia, who also accumulate a large number of risk factors for cervical cancer as young age of marriage, early sexual activity and a high number of pregnancies [32]. The sessions should ensure not to stigmatize less educated women by exposing their ignorance [26]. Trainers should also avoid causing cancer anxiety among women as observed after the launch of an awareness and screening program In Khon Kaen, Thailand [33].

However, even well-conducted, an health education campaign will remain ineffective if the means of secondary prevention are lacking. Counseling and screening for cervical cancer should be included in the National HIV/AIDS programs [34] and implemented in all HIV treatment centers [27]. Knowing the high prevalence of SIL in HIV-infected women, the screening cannot be targeted on the basis of clinical considerations [35] but has to become routine from the age of 25 years. Where cytological screening is not available, it would be relevant to integrate low cost rapid testing at such as VIA, allowing immediate treatment with cryotherapy within a single “see-and-treat” visit. Then, when the screening service is implemented, it can be extended to all women, regardless of their HIV status [36].

Given the screening tools for cervical cancer currently available (cytology, VIA, HPV DNA), it is up to each country to determine, according to its capacities, the most cost-effective strategy for women living with HIV [37]. In Lao PDR, further studies will determine the screening policy best adapted to the scarcity of resources in view of an increasing incidence of cervical cancer.

## Conclusions

Dying from cervical cancer is not a fatality in the karma of Lao women whose vulnerability is primarily due to their ignorance of the HR-HPV infection and its consequences. Despite they are significantly more aware, women living with HIV who bear additional risk of cervical cancer, still have a very poor understanding of the means to prevent it. They should be better informed by the health personnel and be offered a more systematic access to testing.

The implementation of routine screening for cervical cancer in HIV treatment centers would be a significant first step toward a national prevention program aiming at the elimination of a deadly but preventable cancer.

## Competing interests

The authors declare that they have no competing interests.

## Authors’ contributions

CS, FQ, CL and YB contributed to the study design; CS led the entire field survey; CS, FQ, JD and YB performed the statistical analysis; PC and VL gathered valuable information on screening programs in Lao PDR and helped to develop the recommendations. All authors read and approved the final manuscript.

## Pre-publication history

The pre-publication history for this paper can be accessed here:

http://www.biomedcentral.com/1471-2407/14/161/prepub

## Supplementary Material

Additional file 1 Questionnaire. Click here for file
