# Supplementary material for: Knowledge, awareness and attitudes about cervical cancer among women attending or not an HIV treatment center in Lao PDR
Source: BMC Cancer. 2014 Mar 6;14:161. doi: 10.1186/1471-2407-14-161 (PMC4016287; doi:10.1186/1471-2407-14-161)
Supplement: Supplementary file 1 [file 1471-2407-14-161-S1.odt]

Questionnaire

I. Sociodemographics

| No | Questions | Answers |
| --- | --- | --- |
| 1 | Age | … years |
| 2 | Religion | 1 Buddhist, 2 Animist, 3 Christian, 4 other:…… |
| 3 | Marital status | 1 single, 2 bride/concubine, 3 divorced/widowed |
| 4 | Education level | 0 illiterate, 1 primary, 2 secondary, 3 technical college, 4 university, 6 postgraduate |
| 5 | Occupation | 0 housewife, 1 civil servant, 2 shopkeeper, 3 farmer,   4 factory woman, 5 other: … |
| 6 | Monthly income | … USD |

   

II. Status of HIV-infected women 

| No | Data | Results |
| --- | --- | --- |
| A | Year of diagnosis of HIV infection | …. |
| B | CD4 cell count | … / mm3 |
| C | HIV viral load | … copies / ml |
| D | Opportunistic infections | 0: no; 1: yes (specify…) |
| E | Antiretroviral therapy | 0: no; 1: yes (specify compliance) |

   

III. Risk behaviors for sexually transmitted infections

| No | Questions | Answers |
| --- | --- | --- |
| 7 | Have you ever had sex? | 0: no (skip to question 14); 1: yes |
| 8 | Age at first sex | … years |
| 9 | How many children do you have? | ... children |
| 10 | Do you practice a method of contraception? | 0: no (skip to question 13); 1: yes (specify : 1 oral or injectable contraception, 2 coitus interruptus, 3 intra-uterine device, 4 tubal ligation, 5 male or female condom, 6 other …) |
| 11 | If you are taking oral contraceptives, since when do you use? | … years |
| 12 | If you use a condom, how often? | 1 sometimes, 2 often, 3 always |
| 13 | How many sexual partners have you had? | … |
| 14 | Have you smoked? | 0: no; 1: yes (number of pack years) |
| 15 | Have you ever had an STI? | 0: no; 1: yes (specify) ; 2 : no remember |
| 16 | Have you ever consulted a gynecologist? If yes, how often? If not, why? | 0: no; 1: yes … … |
| 17 | Have you ever had a smear test? If so, how often? If not, why?: | 0: no; 1: yes … … |
| 18 | Have you ever had treatment for cervical lesions? | 0: no; 1: yes (specify: 1 conization, 2 laser, 3 hysterectomy, 4 do not know) |

   

IV. Knowledge on cervical cancer and its prevention

| No | Questions | Answers |
| --- | --- | --- |
| 19 | Do you know of STIs? | 0: no; 1: yes (which ones? …) |
| 20 | Have you heard of cancer of the cervix? | 0: no; 1: yes (from what sources? …) |
| 21 | Cervical cancer is a serious illness? Why? | 0: no; 1: yes (specify…); 3: do not know |
| 22 | Do you know the cause of cervical cancer? | 0: no; 1: yes (specify…) |
| 23 | Do you know risk factors for cervical cancer? | 0: no; 1: yes (specify…) |
| 24 | Do you know how to get tested for cervical cancer screening? | 0: no; 1: yes (specify…) |
| 25 | Do you know any methods to prevent cervical cancer? | 0: no; 1: yes (specify…) |
| 26 | Are the women who have had many partners at increased risk of cervical cancer? | 0: no; 1: yes; 3: do not know |
| 27 | Do you know someone with cervical cancer? | 0: no; 1: yes |
| 28 | Do you know what are genital warts? | 0: no; 1: yes (specify…) |
| 29 | Have you heard of human papillomavirus (HPV)? | 0: no; 1: yes (from what sources? …) |
| 30 | Do you know the mode of transmission of HPV? | 0: no; 1: yes (specify…) |
| 31 | Have you heard of the vaccine against HPV? | 0: no; 1: yes (from what sources? …) |

III. Attitudes towards prevention and screening

| No | Questions | Answers |
| --- | --- | --- |
| 32 | Do you think that cervical cancer is a common disease in Laos? | 0: no; 1: yes; 3: do not know |
| 33 | Do you feel at risk of developing cervical cancer? | 0: no; 1: yes (specify…) |
| 34 | Do you think that HIV positive women are more likely to develop cervical cancer? | 0: no; 1: yes; 3: do not know |
| 35 | Do you think that unprotected sex is a risk factor for developing cervical cancer? | 0: no; 1: yes; 3: do not know |
| 36 | Do you think that smoking is a risk factor for developing cervical cancer? | 0: no; 1: yes; 3: do not know |
| 37 | Do you think that multiple sexual partners are a risk factor for developing cervical cancer? | 0: no; 1: yes; 3: do not know |
| 38 | Do you think the government should make recommendations for vaccination against HPV? | 0: no; 1: yes; 3: do not know |
| 39 | Will it need to continue to consult the gynecologist for cervical cancer screening, even after HPV vaccination? | 0: no; 1: yes; 3: do not know |
| 40 | If there were a vaccine against HPV cheap in Laos, would you get vaccinated? | 0: no; 1: yes; 3: do not know |
| 41 | Do you think the HPV vaccine may be given to adolescent girls before the onset of sexual activity? | 0: no; 1: yes; 3: do not know |
| 42 | What do you intend to do to prevent the occurrence of cervical cancer? | 1: take drugs, 2: HPV vaccination, 3 gynecologist examination yearly, 4 : other (specify…) |
